# The Effect of 4-Month Treatment with Glycocalyx Dietary Supplement on Endothelial Glycocalyx Integrity and Vascular Function in Patients with Psoriasis

**DOI:** 10.3390/nu16152572

**Published:** 2024-08-05

**Authors:** Ignatios Ikonomidis, Eleni Katsanaki, John Thymis, George Pavlidis, Kyriaki Lampadaki, Konstantinos Katogiannis, Aristeidis Vaiopoulos, Vicky Lazarou, Gavriella Kostelli, Eleni Michalopoulou, Sotirios Pililis, Dimitrios Vlachomitros, Konstantinos Theodoropoulos, Hans Vink, Robert Long, Evangelia Papadavid, Vaia Lambadiari

**Affiliations:** 1 2nd Cardiology Department, Attikon University Hospital, National & Kapodistrian University of Athens, 12461 Athens, Greece; ignoik@gmail.com (I.I.); helenkatsanaki@gmail.com (E.K.); johnythg@gmail.com (J.T.); geo_pavlidis@yahoo.gr (G.P.); kenndj89@gmail.com (K.K.); kosteligavriela@hotmail.com (G.K.); elenimixa91@gmail.com (E.M.); vlachomitrosdimitrios@gmail.com (D.V.); 2 2nd Department of Dermatology and Venereology, Attikon University Hospital, Medical School, National & Kapodistrian University of Athens, 12461 Athens, Greece; sundylam@yahoo.gr (K.L.); avaiopoulos@gmail.com (A.V.); vicky.lazarou@gmail.com (V.L.); theod28@gmail.com (K.T.); papadavev@yahoo.gr (E.P.); 3 Research Unit and Diabetes Center, 2nd Department of Internal Medicine, Attikon University Hospital, National & Kapodistrian University of Athens, 12461 Athens, Greece; sotiris181@yahoo.gr; 4 GlycoCalyx Research Institute, Alpine, UT 84004, USA; vink@glycocalyx.com (H.V.); long@glycocalyx.com (R.L.)

**Keywords:** glycocalyx, arterial stiffness, pulse wave velocity, augmentation index, fucoidan, glycocalyx dietary supplement, psoriasis

## Abstract

Psoriasis predisposes to cardiovascular dysfunction. We investigated whether glycocalyx dietary supplement (GDS), which contains glycosaminoglycans and fucoidan, improves endothelial glycocalyx and arterial stiffness in psoriatic patients. Fifty participants with psoriasis under biological agents were randomly assigned to GDS (n = 25) or placebo (n = 25) for 4 months. We measured at baseline and at follow-up: (a) perfused boundary region (PBR) of the sublingual microvessels (range 4 to 25 μm), a marker of endothelium glycocalyx integrity; (b) carotid–femoral pulse wave velocity (PWV-Complior SP-ALAM) and augmentation index (AIx), markers of arterial stiffness and (c) psoriasis area and severity index (PASI) score. Both groups displayed a similar decrease in PASI at four months (*p* < 0.05), and no significant differences were found between groups (*p* > 0.05). Compared to the placebo, participants in the GDS showed a greater percentage reduction in PBR4–25 μm (−9.95% vs. −0.87%), PBR 4–9 μm (−6.50% vs. −0.82%), PBR10–19 μm (−5.12% vs. −1.60%), PBR 20–25 μm (−14.9% vs. −0.31%), PWV (−15.27% vs. −4.04%) and AIx (−35.57% vs. −21.85%) (*p* < 0.05). In the GDS group, the percentage reduction in PBR 4–25 μm was associated with the corresponding decrease in PWV (r = 0.411, *p* = 0.015) and AΙx (r = 0.481, *p* = 0.010) at follow-up. Four-month treatment with GDS improves glycocalyx integrity and arterial stiffness in patients with psoriasis. Clinical trial Identifier: NCT05184699.

## 1. Introduction

Psoriasis constitutes a chronic autoimmune disease of the skin yielding increased rates of atherosclerosis, microvascular dysfunction, and impairment of aortic stiffness [1]. Psoriatic patients pose higher cardiovascular risk, as psoriasis shares many pathways with atherosclerosis, including increased oxidative stress burden and inflammation [2,3]. Therefore, psoriasis has been considered as an independent risk factor for cardiovascular events [4,5,6,7].

Endothelial glycocalyx is a structure composed of sulfated proteoglycans, glycoproteins, and glycosaminoglycans that line the luminal side of vascular endothelial cells. It possesses a key role in regulating vascular permeability by preventing leukocytes and platelets from adhering to the endothelium [8,9]. Inflammatory conditions and elevated oxidative stress are associated with glycocalyx degradation, which in turn leads to endothelial dysfunction [10,11]. The degradation of the glycocalyx is speculated to initiate early atherogenic processes and serves as a predictor of adverse cardiovascular outcomes, even in individuals without established cardiac disease [12]. Also, arterial stiffness, which reflects the elastic properties of the aortic wall, is an independent predictor for cardiovascular events [13]. Arterial stiffness has been shown to be affected in chronic inflammatory states, such as in rheumatoid arthritis, systemic lupus erythematous, inflammatory bowel disease [14,15].

Glycocalyx dietary supplement (GDS) constitutes a novel supplement utilized for preservation of the endothelial glycocalyx layer, which aims to improve overall cardiovascular health, enhance blood flow, and reduce the risk of vascular-related conditions [16]. The supplement combines three complementary and synergistic working mechanisms to support the endothelial glycocalyx integrity by supplying sulfated polysaccharides, antioxidant enzymes, and additional substrates for glycocalyx synthesis [17]. Nonetheless, to our knowledge, the effect of GDS on glycocalyx thickness and aortic stiffness in patients with psoriasis, when added to standard treatment with biological agents, has not been elaborated yet.

In view of the former considerations, we aimed to address, prospectively in a case–control study, the effect of GDS on vasculature during a 4-month follow-up period in psoriatic patients commencing biological agents. In particular, we studied whether GDS administration could ameliorate glycocalyx integrity to determine its effect on microvasculature and also endeavored to investigate the impact of GDS on arterial stiffness to determine its effect on the macrovasculature.

## 2. Materials and Methods

### 2.1. Study Population

**Figure 1:**
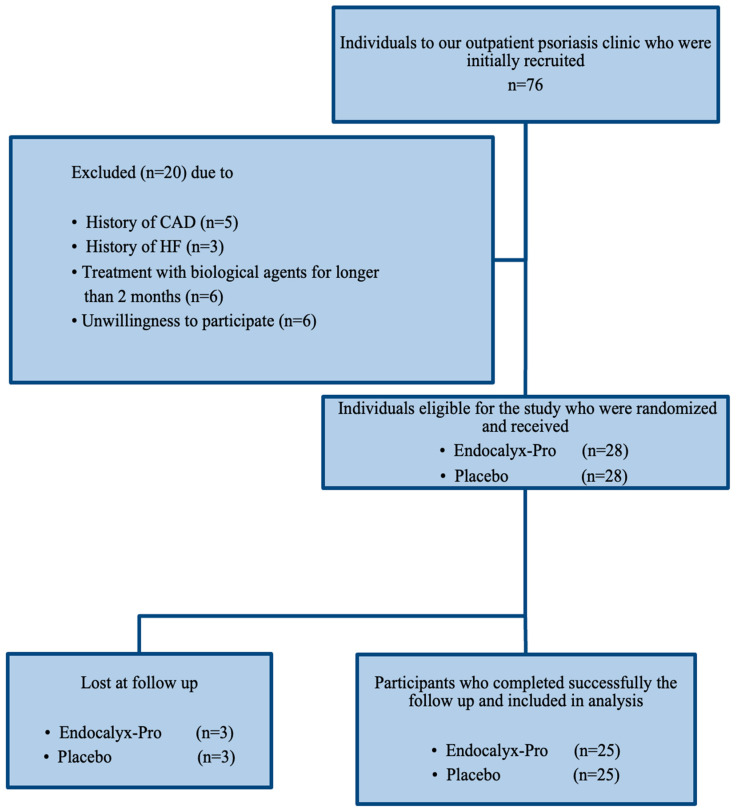
Flowchart of the study progress. CAD, coronary artery disease; HF, heart failure. None of the participants that were lost at follow-up (n = 6) presented at their scheduled appointment in the outpatient psoriasis clinic and could not be contacted via telephone.

We conducted a double-blinded, randomized, placebo-controlled trial to determine the impact of GDS on vascular function and endothelial integrity (Clinical trial.gov Identifier: NCT05184699). Seventy-six psoriatic patients, who were referred to the outpatient clinic of the Dermatology and Venereology Department of Attikon University Hospital, Athens, Greece, were assessed for eligibility by the attending dermatologists. Recruitment occurred from April 2022 to April 2024. Eligible participants were adult patients aged 18 to 75 years old with moderate-to-severe plaque-type psoriasis, who had been commenced on the interleukin (IL)-17A inhibitor secukinumab, the tumor necrosis factor (TNF) inhibitor adalimumab, or the IL23 inhibitor guselkumab within the last 2 months prior to enrollment in the study. Exclusion criteria included pregnancy or breastfeeding, history of coronary artery disease (CAD), moderate or severe valve disease, treatment with biologic agents for more than 2 months, chronic kidney disease (CKD; estimated glomerular filtration rate [eGFR] ≤ 60 mL/min/1.73 m^2^), severe liver disease, and a history of malignancy. Provided that biological agents show beneficial effects on vascular function in as little as 4 months of treatment [1], we decided to exclude patients receiving biological agents more than 2 months before inclusion in the study. Thus, we aimed to secure homogeneity in our sample regarding treatment. Out of the 76 patients, 20 patients were not eligible for the study due to the prevalence of CAD (n = 5), heart failure (n = 3), treatment with biological agents for longer than 2 months (n = 6), and unwillingness to participate in the study (n = 6). Therefore, 56 patients were randomly assigned in the study and received the GDS (n = 28) or placebo (n = 28). The participants received 2 capsules at 8:00 am and 2 capsules at 6:00 pm daily either of the GDS (each serving contains 2556 mg of the blend) or the placebo. The attending dermatologist (E.P.) performed the randomization using a random number table generated by the dedicated online randomization program GraphPad Prism Version 9.3.1 (Dotmatics, Boston, MA, USA) [18], which was accessed on 15 April 2022). Both the patients and the clinicians prescribing the treatment were blinded to the endothelial and vascular function examination results. The follow-up period was set at 4 months. In all participants, we examined endothelial and vascular function at baseline and 4 months after the commencement of the GDS or placebo. The severity of psoriasis was estimated using the psoriasis area and severity index (PASI) score, as indicated [19]. Briefly, the PASI score measures the severity and extent of psoriasis. This index combines the severity of lesions and the area affected into a single score ranging from 0 (no disease) to 72 (maximal disease). Scores below 5 are considered as mild disease, 5–10 as moderate disease, and above 10 as severe disease. Therefore, the PASI score was calculated at both baseline and after treatment to assess its effectiveness. Six participants were lost at follow-up: three from the GDS group and three from the placebo group. None of the participants that were lost at follow-up (n = 6) presented at their scheduled appointment in the outpatient psoriasis clinic and could not be reached via telephone. Hence, 50 participants were finally entailed in the statistical analysis and reported in the study results, 25 in the GDS group and 25 in the placebo group. Figure 1 portrays a comprehensive flowchart of the study progress.

### 2.2. Primary and Secondary Endpoints

The primary outcome of the study was to assess the changes in endothelial glycocalyx integrity and arterial stiffness after 4 months of treatment with GDS compared to the placebo. Secondary outcomes were the comparisons of disease severity between the two study arms at 4-month follow-up.

### 2.3. Composition of the Dietary Supplements

The brand name of the GDS is Endocalyx-Pro. The Endocalyx-Pro supplement is produced and distributed by Microvascular Health Solutions (Alpine, UT, USA). It is a blend (2556 mg per serving of 4 capsules) containing the following: Fucoidan (106.25 mg) extracted from the brown algae species Laminaria japonica, Hyaluronan or hyaluronic acid (17.5 mg); the OxxyneaOMD-MVHS Blend SOD (120 mg) composed of superoxide dismutase (SOD) and polyphenolic enzymes derived from olive, grape, and artichoke; glucosamine sulfate (375 mg), which is obtained from corn; silicon dioxide (2 mg); and microcrystalline cellulose (130 mg).

The placebo capsule mimics the Endocalyx-Pro supplement capsules. It contains 10 mg of the four ingredients Nu-MAG Natural (provided by Ribus (Sparks, NV, USA), IFC Solutions (Linden, NJ, USA), which is composed of rice extract, rice hulls, gum Arabic and sunflower oil. Also, the placebo contains rice flour white (830 mg) and magnesium stearate (10 mg).

At follow-up, no adverse effects were reported regarding the use of Endocalyx-Pro or the placebo.

### 2.4. Imaging of Microcirculation

We evaluated the perfused boundary region (PBR, μm) of the sublingual microvasculature utilizing a sidestream darkfield (SDF) camera (Microscan, GlycoCheck, Microvascular Health Solutions Inc., Salt Lake City, UT, USA) with a diameter range of 4 to 25 μm. Briefly, the SDF camera implements a green reflected light-emitting diode (LED) light (540 nm) to depict the hemoglobin in red blood cells (RBCs) [12]. The camera is placed under the tongue and captures over 3000 microvessel segments [20]. The valid captured images (those with good focus, good contrast, and a minimal level of tissue motion) are saved and analyzed using GlycoCheck software version 5.3.3 [20]. The median RBC column width and the total perfused diameter of the microvessels are assessed using the software. PBR is estimated using the following formula: (perfused diameter − median RBC column width)/2 [20]. Afterwards, the software calculates the PBR of the microvessels with diameter ranging from 4 to 9 μm, 10 to 19 μm, and 20 to 25 μm [20]. A high PBR implies that RBCs penetrate more deeply into the endothelial surface, which represents a compromised glycocalyx layer with affected barrier properties and vice versa [20] (Supplementary Materials). The measurement takes approximately three minutes to complete. The reported variabilities for PBR calculation of the same and between different observers are 4.3% and 5.2%, respectively [21]. Of note, PBR evaluation is independent of hematocrit, as the software encompasses solely vessel segments with red blood cells filling a percentage of more than 50% [20,21].

### 2.5. Arterial Stiffness

The carotid–femoral pulse wave velocity (PWV) is regarded as the gold standard for measuring arterial stiffness [22]. According to the European Society of Hypertension (ESH) guidelines for managing arterial hypertension, PWV values over 10 m/s in hypertensive individuals suggest increased arterial stiffness with altered properties of the aortic wall, which indicates altered wall properties [23,24]. To calculate PWV, we employed the direct method to measure the distance between the carotid and femoral artery using the Complior device (Alam Medical, Vincennes, France). Then, we implemented the appropriate corrections by multiplying the default PWV values with 0.8 [22].

The augmentation index (AIx, %) was measured using an arm cuff with oscillometry, as previously published. During systole, the blood volume ejected into the aorta creates an initial pulse wave (early systolic peak, P1). This pulse wave travels down the aorta, reflects at the bifurcation, and forms a second wave (late systolic peak, P2). Both the early and late systolic peaks were recorded as pulse waves on the computer. AIx (%) was calculated using the formula [(P2 − P1)/pulse pressure (PP)] × 100 [25].

### 2.6. Statistical Analysis

Statistical analysis was conducted using SPSS version 29 (IBM SPSS Statistics, Inc., Chicago, IL, USA). All scale variables were presented as mean ± SD in case of normal distribution. The Kolmogorov–Smirnov and Shapiro–Wilk normality tests were applied to determine the distribution. When normality criteria were not met, we transformed the variables into ranks. Categorical variables were presented as absolute and relative frequencies. We conducted Spearman or Pearson correlation tests, as appropriate, for correlation analysis. Nominal variables were examined using either Chi-square tests or Fisher’s exact tests, as appropriate. All the analyses were conducted with the intention to treat. Analysis of variance (ANOVA) for repeated measurements was implemented for (1) comparisons of the markers of vascular function, endothelial integrity, and disease severity as well (2) the effects of the type of intervention, as a between-subject factor (the 2 intervention arms consisted of the GDS group and the placebo group). The F and corresponding *p* values for the change in the markers at follow-up measurement were estimated. Also, the F and *p* values for the effect between time of measurement and the intervention arms were calculated. When Mauchly’s test indicated that the sphericity assumption was not satisfied, corrections such as Greenhouse–Geisser, Huynh–Feldt, or lower bound correction were applied. All statistical tests were conducted as two-tailed, with a *p*-value of less than 0.05 deemed statistically significant.

## 3. Results

The population characteristics are provided in Table 1. Briefly, the mean age of the population was 54.5 ± 8.8 years, and 22/50 (44%) were females. Additionally, the mean PASI score was 13 ± 3.8, indicating that all participants had moderate-to-severe disease, and the mean duration of psoriasis was 16.3 ± 10 years. Of note, approximately 17/50 (34%) were suffering from hypertension and 16/50 (32%) from dyslipidemia, and 23/50 (46%) were current smokers at inclusion. Also, 19/50 (38%) participants were receiving IL-17 inhibitors, 15/50 (30%) TNF inhibitors, and 16/50 (32%) IL-23 inhibitors. Comparisons of the baseline characteristics between the two groups were non-significant (*p* > 0.05). At baseline, increased PASI was associated with elevated PBR4–25 (r = 0.347) and PWV (r = 0.311) in all patients (*p* < 0.05 for all comparisons).

### 3.1. Control of Psoriasis after the 4-Month Intervention

Baseline PASI did not differ between the two groups (*p* > 0.05). At 4-month follow-up, all participants achieved a significant reduction in PASI at 4 months from 13 ± 3.8 to 2.2 ± 0.8, which indicates mild disease (F = 35.30; *p* < 0.001). No significant differences were recorded between the two groups regarding PASI at follow-up (for the GDS group: 84.61%; 95% CI: −87.32% to −82.13. For the placebo group; 95% CI: −87.32% (*p* > 0.05)) (Table 2).

### 3.2. Change in Thickness of Endothelial Glycocalyx

Baseline PBR values did not differ significantly between the two groups (*p* > 0.05). In total, PBR4–25 decreased significantly at the 4-month follow-up from 2.30 ± 0.22 μm to 2.17 ± 0.26 μm (F = 8.32; *p* = 0.003). Of note, we observed a significant interaction between the intervention arm and the change in PBR4–25 at follow-up (F = 6.97; *p* = 0.009) (Table 2). More specifically, participants in the GDS group demonstrated a significant reduction in PBR4–25 (−9.95%; 95%CI: −10.34 to −8.35%) compared to the placebo group (−0.87%; 95%CI: −2.34–1.64%).

A similar trend was noticed in PBR 4–9, 10–19, and 20–25, respectively (Table 2).

### 3.3. Change in Arterial Stiffness

Baseline PWV values did not differ significantly between the two groups (*p* > 0.05). PWV improved remarkably at follow-up in the study population from 10.40 ± 2.14 m/s to 9.39 ± 2.23 m/s (F = 9.75; *p* = 0.002). Additionally, we noted an interaction between the intervention arm and the change in PWV at follow-up (F = 7.56, *p* = 0.004). Participants in the GDS group displayed a greater reduction in PWV compared to the placebo group (−15.27%; 95%CI: −18.48 to −13.65% versus −4.04%; 95%CI: −6.68 to −1.32%, respectively).

Baseline AIx values did not differ significantly between the two groups (*p* > 0.05). AIx reduced remarkably at follow-up in the study population from 12 ± 4.12% to 8.56 ± 3.23 m/s (F = 19.70; *p* < 0.001). Additionally, we noted an interaction between the intervention arm and the change in AIx at follow-up (F = 4.02, *p* = 0.037). Participants in the GDS group showed a greater reduction in AIx compared to the placebo group (−35.57%; 95%CI: −40.89 to −31.14% versus −21.85%; 95%CI: −26.44 to −17.42%, respectively).

### 3.4. Associations between Glycocalyx Integrity, Arterial Stiffness, and Psoriasis Severity at 4-Month Follow-Up

In the total sample, the percentage change in PASI was correlated with the respective percentage change in PBR4–25 (r = 0.390, *p* = 0.019) and PWV (r = 0.335, *p* = 0.020).

In the GDS group, the percentage change in PBR 4–25 was associated with the percentage change in PWV (r = 0.411, *p* = 0.015) and AIx (r = 0.481, *p* = 0.010), correspondingly.

## 4. Discussion

In the current study, we showed that patients with psoriasis treated with the GDS for 4 months as an add-on treatment to the standard treatments with biological agents displayed remarkable improvement in endothelial glycocalyx thickness of microvasculature in conjunction with substantial attenuation of arterial stiffness compared with the placebo group. In particular, participants under GDS demonstrated a greater reduction in PBR compared with the placebo group. Additionally, participants in the GDS group achieved greater improvement in both PWV and AIx compared with the placebo group. Notably, in the GDS group, the improvement in PBR was associated with the decrease in PWV and AIx. Consequently, GDS administration for a short-term period seems to exert robust effects in both endothelial integrity and arterial stiffness, and these effects occur concurrently.

### 4.1. Effects of GDS on Glycocalyx Integrity

In the current study, we showed that GDS administration resulted in profound improvement in glycocalyx integrity, as assessed by PBR measurement. We have previously shown that patients with psoriasis demonstrate affected PBR values compared to healthy controls, which is mainly attributed to the increased inflammatory burden and increased oxidative stress burden [21].

Indeed, dysregulation of the glycocalyx layer occurs in patients with chronic inflammatory conditions like psoriasis [26]. Inflammation-mediated depletion of the endothelial glycocalyx by pro-inflammatory cytokines such as (TNF)-a IL-1, 6, 17A, and 8 promote changes in endothelial permeability, resulting in increased transcapillary water and protein permeability [1,26,27]. These in turn result in a shift of interstitial fluid, and edema finally ensues [1,26,27]. Also, in chronic inflammatory states, high plasma concentrations of syndecan-1 and heparan sulfate, which are major components of the glycocalyx layer, are found [28]. These findings indicate that inflammatory processes directly induce the degradation of the glycocalyx layer [28].

Interestingly, in the current study, participants in the placebo group displayed a slight non-significant improvement in PBR compared to baseline, suggesting that biological treatment may also have an effect on endothelial glycocalyx through a reduction in the inflammatory process. However, the addition of GDS to biological treatment causes a greater improvement in endothelial glycocalyx compared to the placebo. A possible interpretation is that GDS contains glycocalyx substrates, such as hyaluronic acid; fucoidan, which is considered a heparinase inhibitor; antioxidants, such as superoxide dismutase (SOD); and polyphenolic enzymes, which protect against damage from free radicals and other ingredients that may assist in the restoration of the depleted glycocalyx layer [29,30]. In particular, fucoidan constitutes a fucosylated and sulfated polysaccharide that mimics heparan sulfate, a major glycosaminoglycan of the endothelial glycocalyx [29,30]. Hyaluronan is another major glycosaminoglycan present in the glycocalyx layer [29,30]. Another component of the GDS includes glucosamine sulfate, which is implicated in the synthesis of key components of the endothelial glycocalyx [29,30]. A recently published study conducted in chronic kidney disease patients showed that GDS resulted in the restoration of glycocalyx integrity, as measured by atomic force microscopy [29]. In the same study, the effectiveness of GDS was also addressed by reporting a drop in the PBR of mice during in vivo measurement [29]. It was speculated that GDS’s effects are possibly mediated by the mitogen-activated protein kinase (MAPK)/extracellular-signal-regulated kinase (ERK) and phosphatidylinositol 3-kinase (PI3K) signaling pathways [29]. In another recent study, 10 weeks of dietary glycocalyx precursor supplementation in old mice resulted in restoration of glycocalyx barrier function accompanied by attenuation of aortic stiffness and augmented endothelium-dependent dilation and nitric oxide bioavailability, suggesting that glycocalyx may be an effective therapeutic target for vascular dysfunction in older adults [17]. Their findings regarding improvement in PBR are aligned with ours. However, there are crucial differences between the two study populations. Another study in patients with COVID-19, who are also considered to exhibit endothelial impairment due to inflammation, reported restoration of the disrupted glycocalyx following administration of fucoidan [31]. Similarly, data obtained from diabetic patients indicate that GDS administration significantly reduced PBR while also lowering HbA1c levels [32]. In contrast, a study conducted in older adults was inconclusive regarding the role of GDS on endothelial glycocalyx, as there was no difference in PBR4–25 between the GDS group and placebo group at 3-month follow-up. Interestingly, in the above study, a subgroup analysis of participants who were not treated with antihypertensive agents revealed a significant improvement in PBR of microvessels ranging from 4 to 7 μm in the GDS group [33]. Nonetheless, our study is the first to address the effect of GDS in psoriasis patient treatment. Its robust effects on the glycocalyx layer appear to be attributed to its ingredients, such as fucoidan, which counterbalance the detrimental effects of chronic inflammation and restore the glycocalyx layer. Therefore, GDS may be used in conjunction with standard treatment of psoriasis to enhance its effectiveness on endothelial function.

### 4.2. Effects of GDS on Arterial Stiffness

We documented a significant decrease in both PWV and AIx in participants treated with GDS, as an add-on treatment to standard treatment with biological agents, compared to placebo. Patients with psoriasis exert affected stiffness properties owing to persistent inflammation, increased oxidative stress burden, and a higher prevalence of the conventional risk factors for cardiovascular disease [34]. We have previously shown that biological agents reduced PWV in patients with psoriasis [1]. Of note, we observed that GDS administration led to greater improvement in PWV and AIx when added to standard treatment with biological agents. Additionally, a significant association between the change in PBR and PWV and AIx was also documented.

Taking into consideration the above findings, we speculate that the favorable effect of GDS on arterial stiffness is explained by its beneficial effect on endothelial function. In support of this hypothesis, an experimental study showed that GDS treatment resulted in improvement in PBR, which was in parallel with the drop in PWV in mice [16]. The proposed pathophysiologic mechanisms include that a thicker glycocalyx layer promotes more efficient shear stress transduction and endothelial-dependent nitric oxide (NO) release, which in turn ameliorate arterial wall stiffness [16]. Thus, GDS appears to aggravate the effects of biological agents on arterial stiffness indirectly through improvement in endothelial function.

The following limitations also apply to our study. Firstly, the sample size is relatively small and, therefore, a larger cohort with longer follow-up period is warranted to validate our findings. Secondly, we addressed only the aortic stiffness and the thickness of glycocalyx layer of the microvasculature. Given that psoriasis also impairs cardiac function, in the future, we could assess whether GDS preserves or improves cardiac function in psoriatic patients. Hence, further studies are required to investigate the effect of this supplement on the top of other treatments of psoriasis.

The current study is a well-designed double-blinded, randomized, placebo-controlled trial and provides novel evidence for the effects of GDS on microvascular function and arterial elastic properties.

## 5. Conclusions

In conclusion, in the present study, we demonstrated that the addition of GDS to biological treatments resulted in robust effects in the vasculature, albeit all participants achieved significant control of psoriasis, as reflected by the reduction in PASI score. More specifically, patients receiving GDS displayed substantial improvement in PBR, a metric of sublingual microvessel thickness, in concordance with the amelioration of PWV, a metric of aortic stiffness. These findings suggest that GDS could be included in the management of psoriasis to effectively reduce cardiovascular disease burden.

## Figures and Tables

**Table 1:** Baseline characteristics of the study population.

	All Participants (N = 50)	GDS (N = 25)	Placebo (N = 25)	*p*-Value
Age (years)	54.5 ± 8.8	53.4 ± 9	55.6 ± 8.8	0.481
Sex (male/female)	28/22	13/12	15/10	0.777
PASI score	13 ± 3.8	12.8 ± 3.6	13.2 ± 3.9	0.512
Disease duration (years)	16.3 ± 10	17.3 ± 10.9	15.3 ± 9.2	0.368
Risk factors, n (%)				
Current smoking	23 (46)	13 (52)	10 (40)	0.394
Diabetes mellitus	9 (18)	4 (16)	5 (20)	0.712
Dyslipidemia	16 (32)	10 (40)	6 (24)	0.225
Family history of CAD	10 (20)	4 (16)	6 (24)	0.479
Hypertension	17 (34)	7 (28)	10 (40)	0.370
Medications, n (%)				
ACEi/ARBs	15 (30)	6 (24)	9 (36)	0.354
Antidiabetic agents	9 (18)	4 (16)	5 (20)	0.712
Beta blockers	5 (10)	2 (8)	3 (12)	0.637
CCBs	10 (20)	3 (12)	7 (28)	0.157
Diuretics	11 (22)	3 (12)	8 (32)	0.090
Statins	16 (32)	10 (40)	6 (24)	0.225
Biological agents				0.369
IL-17 inhibitors	19 (38)	10 (40)	9 (36)	
IL-23 inhibitors	16 (32)	7 (28)	9 (36)	
TNF inhibitors	15 (30)	8 (32)	7 (28)	
SBP (mm Hg)	134.3 ± 18.9	131.1 ± 16.4	137.5 ± 20.3	0.272
DBP (mm Hg)	81.4 ± 11.9	79.8 ± 7.9	83.1 ± 10.2	0.207
HbA1C (%)	6.6 ± 0.9	6.8 ± 1	6.4 ± 0.8	0.130
Total cholesterol (mg/dL)	196.5 ± 40.9	203.1 ± 38	190.5 ± 42.2	0.273
LDL-C (mg/dL)	129.3 ± 32.9	134.1 ± 29.5	125.5 ± 35.1	0.353
HDL-C (mg/dL)	47.1 ± 16.7	48.4 ± 15.9	46.5 ± 17.4	0.688
Triglycerides (mg/dL)	101.4 ± 13.8	104 ± 11.9	98.2 ± 15.7	0.106

Data are presented as absolute frequencies (relative frequencies), mean ± SD, or median (first quartile to third quartile). Scale variables were compared using Student’s *t*-test. Binary variables were compared using the χ^2^ test. ACEI, angiotensin-converting enzyme inhibitor; ARB, angiotensin receptor blocker; CAD, coronary artery disease; CCB, calcium channel blocker; DBP, diastolic blood pressure; GDS, glycocalyx dietary supplement; HbA1c, glycosylated hemoglobin; HDL-C, high-density lipoprotein cholesterol; IL, interleukin; SBP; systolic blood pressure, PASI, psoriasis area severity index; TNF, tumor necrosis factor.

**Table 2:** Changes in glycocalyx thickness and arterial stiffness at 4 months.

	All Participants (n = 50)	GDS (n = 25)	Placebo (n = 25)
PBR4–25 (μm)			
Baseline	2.30 ± 0.22	2.31 ± 0.20	2.29 ± 0.25
4 months	2.17 ± 0.26 ^††^	2.08 ± 0.31 ^††,^**	2.27 ± 0.23
Δ%	−5.65	−9.95	−0.87
PBR4–9 (μm)			
Baseline	1.23 ± 0.26	1.23 ± 0.30	1.22 ± 0.23
4 months	1.18 ± 0.21 ^†^	1.15 ± 0.18 ^††,^*	1.21 ± 0.24
Δ%	−4.06	−6.50	−0.82
PBR10–19 (μm)			
Baseline	2.52 ± 0.28	2.54 ± 0.26	2.50 ± 0.31
4 months	2.43 ± 0.31 ^††^	2.41 ± 0.35 ^††,^*	2.46 ± 0.27
Δ%	−3.57	−5.12	−1.60
12 mo			
PBR20–25 (μm)			
Baseline	3.15 ± 0.34	3.15 ± 0.34	3.15 ± 0.33
4 months	2.91 ± 0.29 ^†††^	2.68 ± 0.30 ^†††,^***	3.14 ± 0.29
Δ%	−7.62	−14.9	−0.31
PWV (m/s)			
Baseline	10.40 ± 2.14	10.41 ± 2.11	10.39 ± 2.24
4 months	9.39 ± 2.23 ^††^	8.82 ± 2.29 ^††,^**	9.97 ± 2.17 ^†^
Δ%	−9.71	−15.27	−4.04
AIx (%)			
Baseline	12 ± 4.12	11.92 ± 4.08	12.08 ± 4.16
4 months	8.56 ± 3.23 ^††^	7.68 ± 3.12 ^††,^*	9.44 ± 3.34 ^†^
Δ%	−28.66	−35.57	−21.85
PASI score			
Baseline	13 ± 3.8	12.8 ± 3.6	13.2 ± 3.9
4 months	2.2 ± 0.8 ^†††^	2 ± 0.6 ^†††^	2.4 ± 1 ^†††^
Δ%	−82.67	−84.61	−81.95

Data are expressed as mean values ± SD. Δ%: percent changes from baseline. AIx, augmentation index; GDS, glycocalyx dietary supplement; PASI, psoriasis area severity index; PBR, perfused boundary region of sublingual microvessels with diameter range of 4–25 μm, 4–9 μm, 10–19 μm and 20–25 μm; PWV, pulse wave velocity. * *p* < 0.05; ** *p* < 0.01; *** *p* < 0.001 for time × treatment interaction obtained by repeated measures ANOVA. ^†^ *p* < 0.05, ^††^ *p* < 0.01, ^†††^ *p* < 0.001 for comparisons of 4 months versus baseline by ANOVA.

## Data Availability

The original contributions presented in the study are included in the article/Supplementary Materials, further inquiries can be directed to the corresponding author.

## Supplementary Materials

The following supporting information can be downloaded at https://www.mdpi.com/article/10.3390/nu16152572/s1: Figure S1: Measurement of Perfused Boundary Region by Glycocheck.

## References

[1] Makavos G., Ikonomidis I., Andreadou I., Varoudi M., Kapniari I., Loukeri E., Theodoropoulos K., Pavlidis G., Triantafyllidi H., Thymis J. (2020). Effects of interleukin 17A inhibition on myocardial deformation and vascular function in psoriasis. Can. J. Cardiol..

[2] Greb J.E., Goldminz A.M., Elder J.T., Lebwohl M.G., Gladman D.D., Wu J.J., Mehta N.N., Finlay A.Y., Gottlieb A.B. (2016). Psoriasis. Nat. Rev. Dis. Primers.

[3] Garshick M.S., Ward N.L., Krueger J.G., Berger J.S. (2021). Cardiovascular risk in patients with psoriasis: JACC Review Topic of the Week. J. Am. Coll. Cardiol..

[4] Gelfand J.M., Neimann A.L., Shin D.B., Wang X., Margolis D.J., Troxel A.B. (2006). Risk of myocardial infarction in patients with psoriasis. JAMA.

[5] Horreau C., Pouplard C., Brenaut E., Barnetche T., Misery L., Cribier B., Jullien D., Aractingi S., Aubin F., Joly P. (2013). Cardiovascular morbidity and mortality in psoriasis and psoriatic arthritis: A systematic literature review. J. Eur. Acad. Dermatol. Venereol..

[6] Raaby L., Ahlehoff O., de Thurah A. (2017). Psoriasis and cardiovascular events: Updating the evidence. Arch. Dermatol. Res..

[7] Elmets C.A., Leonardi C.L., Davis D.M.R., Gelfand J.M., Lichten J., Mehta N.N., Armstrong A.W., Connor C., Cordoro K.M., Elewski B.E. (2019). Joint AAD-NPF guidelines of care for the management and treatment of psoriasis with awareness and attention to comorbidities. J. Am. Acad. Dermatol..

[8] Lekakis J., Abraham P., Balbarini A., Blann A., Boulanger C.M., Cockcroft J., Cosentino F., Deanfield J., Gallino A., Ikonomidis I. (2011). Methods for evaluating endothelial function: A position statement from the European Society of Cardiology Working Group on Peripheral Circulation. Eur. J. Cardiovasc. Prev. Rehabil..

[9] Yilmaz O., Afsar B., Ortiz A., Kanbay M. (2019). The role of endothelial glycocalyx in health and disease. Clin. Kidney J..

[10] Nieuwdorp M., Meuwese M.C., Vink H., Hoekstra J.B., Kastelein J.J., Stroes E.S. (2005). The endothelial glycocalyx: A potential barrier between health and vascular disease. Curr. Opin. Lipidol..

[11] Mulders T.A., Nieuwdorp M., Stroes E.S., Vink H., Pinto-Sietsma S.J. (2013). Non-invasive assessment of microvascular dysfunction in families with premature coronary artery disease. Int. J. Cardiol..

[12] Ikonomidis I., Thymis J., Simitsis P., Koliou G.A., Katsanos S., Triantafyllou C., Kousathana F., Pavlidis G., Kountouri A., Polyzogopoulou E. (2022). Impaired Endothelial Glycocalyx Predicts Adverse Outcome in Subjects without Overt Cardiovascular Disease: A 6-Year Follow-up Study. J. Cardiovasc. Transl. Res..

[13] Mitchell G.F., Hwang S.-J., Vasan R.S., Larson M.G., Pencina M.J., Hamburg N.M., Vita J.A., Daniel Levy D., Benjamin E.J. (2010). Arterial stiffness and cardiovascular events: The Framingham Heart Study. Circulation.

[14] Laurent S., Cockcroft J., Bortel L.V., Boutouyrie P., Giannattasio C., Hayoz D., Pannier B., Vlachopoulos C., Wilkinson I., Struijker-Boudier H. (2006). Expert consensus document on arterial stiffness: Methodological issues and clinical applications. Eur. Heart J..

[15] Triantafyllou C., Nikolaou M., Ikonomidis I., Bamias G., Kouretas D., Andreadou I., Tsoumani M., Thymis J., Papaconstantinou I. (2021). Effects of Anti-Inflammatory Treatment and Surgical Intervention on Endothelial Glycocalyx, Peripheral and Coronary Microcirculatory Function and Myocardial Deformation in Inflammatory Bowel Disease Patients: A Two-Arms Two-Stage Clinical Trial. Diagnostics.

[16] Machin D.R., Trott D.W., Gogulamudi V.R., Islam M.T., Bloom S.I., Vink H., Lesniewski L.A., Donato A.J. (2023). Glycocalyx-targeted therapy ameliorates age-related arterial dysfunction. Geroscience.

[17] Machin D.R., Nguyen D., Bramwell R.C., Lesniewski L.A., Donato A.J. (2019). Dietary glycocalyx precursor supplementation ameliorates age-related vascular dysfunction. FASEB J..

[18] GraphPad: Home. https://www.graphpad.com/.

[19] Langley R.G., Ellis C.N. (2004). Evaluating psoriasis with Psoriasis Area and Severity Index, Psoriasis Global Assessment, and Lattice System Physician’s Global Assessment. J. Am. Acad. Dermatol..

[20] Lee D.H., Dane M.J.C., van den Berg B.M., Boels M.G.S., van Teeffelen J.W., de Mutsert R., den Heijer M., Rosendaal F.R., van der Vlag J., Jan van Zonneveld A. (2014). Deeper penetration of erythrocytes into the endothelial glycocalyx is associated with impaired microvascular perfusion. PLoS ONE.

[21] Ikonomidis I., Pavlidis G., Lambadiari V., Rafouli-Stergiou P., Makavos G., Thymis J., Kostelli G., Varoudi M., Katogiannis K., Theodoropoulos K. (2021). Endothelial glycocalyx and microvascular perfusion are associated with carotid intima-media thickness and impaired myocardial deformation in psoriatic disease. J. Hum. Hypertens..

[22] Varoudi M., Andreadou I., Gravanis K., Theodoropoulos K., Pavlidis G., Triantafyllidi H., Parissis J., Paraskevaidis I., Rigopoulos D., Lekakis J. (2015). Similarities in coronary function and myocardial deformation between psoriasis and coronary artery disease: The role of oxidative stress and inflammation. Can. J. Cardiol..

[23] Kapuku G.K., Harshfield G.A., Davis H.C., Treiber F.A. (2006). Early markers of cardiovascular disease Vascular Pharmacology. Vasc. Pharmacol..

[24] Mancia G., Kreutz R., Brunström M., Burnier M., Grassi G., Januszewicz A., Muiesan M.L., Tsioufis K., Agabiti-Rosei E., Algharably E.A.E. (2023). 2023 ESH Guidelines for the management of arterial hypertension The Task Force for the management of arterial hypertension of the European Society of Hypertension: Endorsed by the International Society of Hypertension (ISH) and the European Renal Association (ERA). J. Hypertens..

[25] Ikonomidis I., Marinou M., Vlastos D., Kourea K., Andreadou I., Liarakos N., Triantafyllidi H., Pavlidis G., Tsougos E., Parissis J. (2017). Effects of varenicline and nicotine replacement therapy on arterial elasticity, endothelial glycocalyx and oxidative stress during a 3-month smoking cessation program. Atherosclerosis.

[26] Chelazzi C., Villa G., Mancinelli P., De Gaudio A.R., Adembri C. (2015). Glycocalyx and sepsis-induced alterations in vascular permeability. Crit. Care.

[27] Tanaka T., Narazaki M., Kishimoto T. (2016). Immunotherapeutic implications of IL-6 blockade for cytokine storm. Immunotherapy.

[28] Franceković P., Gliemann L. (2023). Endothelial Glycocalyx Preservation-Impact of Nutrition and Lifestyle. Nutrients.

[29] Regier M., Drost C.C., Rauen M., Pavenstädt H., Rovas A., Kümpers P., Vink H., Long R.M., Linke W.A., Nofer J.R. (2022). A Dietary Supplement Containing Fucoidan Preserves Endothelial Glycocalyx through ERK/MAPK Signaling and Protects against Damage Induced by CKD Serum. Int. J. Mol. Sci..

[30] Li X., Li X., Zhang Q., Zhao T. (2017). Low molecular weight fucoidan and its fractions inhibit renal epithelial mesenchymal transition induced by TGF-beta1 or FGF-2. Int. J. Biol. Macromol..

[31] Yuan L., Cheng S., Sol W.M.P.J., van der Velden A.I.M., Vink H., Rabelink T.J., van den Berg B.M. (2022). Heparan sulfate mimetic fucoidan restores the endothelial glycocalyx and protects against dysfunction induced by serum of COVID-19 patients on ICU. ERJ Open Res..

[32] van der Velden A.I.M., IJpelaar D.H.T., Chandie Shaw P.K., Pijl H., Vink H., van der Vlag J., Rabelink T.J., van den Berg B.M. (2024). Role of dietary interventions on microvascular health in South-Asian Surinamese people with type 2 diabetes in the Netherlands: A randomized controlled trial. Nutr. Diabetes.

[33] Gimblet C.J., Ernst J.W., Bell B., Bos K.D., Stroud A.K., Wendt L.H., Donato A.J., Jalal D.I., Pierce G.L. (2024). Effect of glycocalyx-targeted therapy on vascular function in older adults: A randomized controlled trial. J. Appl. Physiol..

[34] Gisondi P., Fantin F., Del Giglio M., Valbusa F., Marino F., Zamboni M., Girolomoni G. (2009). Chronic plaque psoriasis is associated with increased arterial stiffness. Dermatology.

