# Supplementary material for: The Effect of 4-Month Treatment with Glycocalyx Dietary Supplement on Endothelial Glycocalyx Integrity and Vascular Function in Patients with Psoriasis
Source: Nutrients. 2024 Aug 5;16(15):2572. doi: 10.3390/nu16152572 (PMC11313920; doi:10.3390/nu16152572)
Supplement: Supplementary file 1 [file nutrients-16-02572-s001.zip › nutrients-3144321-supplementary.docx]

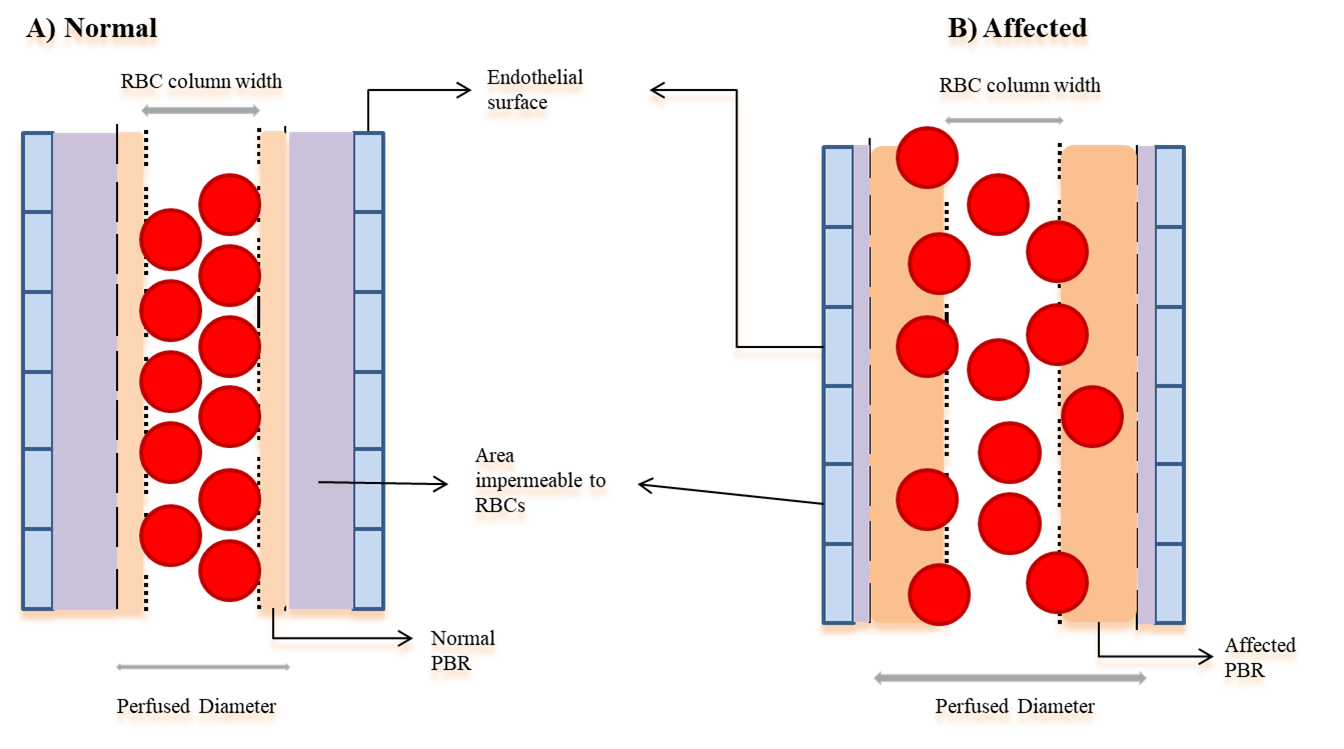


**Supplementary Figure**. Measurement of Perfused Boundary Region by Glycocheck. RBCs, Red Blood Cells; SDF, Sideview Darkfield Imaging; PBR, Perfused Boundary Region . llustration A) depicts the concept of normal non-affected glycocalyx layer. PBR is calculated by the following formula: [Perfused Diameter - RBC column width]/2. llustration B) portrays the concept of glycocalyx with reduced thickness and increased affected PBR resulting in greater than normal lateral displacement of RBCs.
